# Comparing Questionnaires for Assessing Orthorexic Thoughts and Behaviors in College Students

**DOI:** 10.1007/s11414-025-09957-z

**Published:** 2025-07-25

**Authors:** Marta Plichta, Radosław Rogoza

**Affiliations:** 1 Department of Food Market and Consumer Research, Institute of Human Nutrition Sciences, Warsaw University of Life Sciences (SGGW-WULS), Nowoursynowska St. 159C, 02-776 Warsaw, Poland; 2 Department of Human Sciences, University of Economics and Human Sciences in Warsaw, Okopowa St. 59, 01-043 Warsaw, Poland

**Keywords:** Orthorexic thoughts and behaviors, ORTO; PL-DOS, Questionnaires comparison, Diet quality, Mindful eating, College students

## Abstract

Mindful eating (ME) is becoming increasingly popular as a method of changing problematic eating behaviors, including orthorexic (ON) thoughts and behaviors. Simultaneously, the psychometric flaws of commonly used questionnaires for assessing ON thoughts and behaviors limit the potential of exploring their association with ME and diet quality. The study aimed to (a) assess the intensity of ON thoughts and behaviors; (b) evaluate the associations between ON thoughts and behaviors, ME, and diet quality; and (c) compare ORTO-R with ORTO-15 and its shortened versions and PL-DOS. Data were collected from a sample of 478 college students through a cross-sectional study conducted in 2021 across eighteen universities in Poland. Students completed the ORTO-R, ORTO-15, Düsseldorf Orthorexia Scale (PL-DOS), Mindful Eating Scale (MES), and Dietary Habits and Nutrition Beliefs Questionnaire (KomPAN). ORTO-15 and its shortened versions correlated at least highly positively with each other for both sexes, as did ORTO-R and PL-DOS. Positive correlations were found between ORTO-15 and its shortened versions and such mindful eating behaviors as act with awareness as well as awareness for females and/or males. Meanwhile, ORTO-R correlated negatively with act with awareness and acceptance for females and/or males. In addition, PL-DOS correlated negatively with awareness and acceptance for females and/or males. An increase in the intensity of ON thoughts and behaviors measured by almost all questionnaires was associated with an increase in the intensity of beneficial dietary characteristics for health (higher the “Pro-Healthy Diet Index”—pHDI, and the “Diet Quality Index”—DQI) and a decrease in the intensity of harmful dietary characteristics for health (lower the “Non-Healthy Diet Index”—nHDI). Overall, ORTO-R and PL-DOS proved to be the most effective questionnaires for distinguishing eating behaviors in the context of orthorexic thoughts and behaviors, but only for ME. The results may suggest that ME practices may be associated with reduced ON thoughts and behaviors. However, limitations of the study, for example, a sample of college students, a non-randomly selected of sample, a cross-sectional design of the study, and an imbalanced biological sex ratio, make it much more difficult to generalize the results.

**Supplementary Information:**

The online version contains supplementary material available at 10.1007/s11414-025-09957-z.

## Introduction

Orthorexic (ON) thoughts and behaviors describe a compulsive fixation with healthy eating.^1^ Within these thoughts and behaviors, an excessive focus on food quality and purity, strict eating rules, inflexibility in eating, and attempts to achieve the “perfect” diet are observed.^2^ Although such behaviors may lead to serious mental and physical health problems—including psychological and/or social impairment, severe malnutrition, and substantial weight loss—they have not been officially recognized as an eating disorder under the name orthorexia nervosa.^2^ These behaviors are particularly prevalent among college students, especially those studying health, nutrition, and food sciences, as well as among health professionals.^3^

Within the framework of studies assessing ON thoughts and behaviors, several questionnaires have been used, with the ORTO-15 being the most recognized.^4^ However, the psychometric properties of the ORTO-15 have been repeatedly criticized.^5^ To address these issues, various shortened versions of ORTO have been developed by excluding specific items to improve the questionnaire’s face validity, internal consistency, model fit, or factor interpretability.^4^ Despite these efforts, studies have shown that the shortened versions share the same psychometric flaws as the original ORTO-15, and they still fail to differentiate between a genuine interest in healthy eating and a compulsive fixation on healthy eating.^4^ In response to these limitations, new questionnaires have been developed, including the Düsseldorf Orthorexia Scale (DOS),^6^ the Eating Habits Questionnaire (EHQ),^7^ the Teruel Orthorexia Scale (TOS),^8^ and the Orthorexia Nervosa Inventory (ONI).^9^ In addition, Rogoza and Donnini,^10^proposed a revised version of the ORTO-15, known as ORTO-R, which has been validated among Polish populations, including a sample from the current study.^11^ Previous findings confirmed that the Polish version of ORTO-R is a valid and internally consistent tool for evaluating ON thoughts and behaviors.^11^ Using these questionnaires may help advance the ongoing debate about the pathological relevance of these behaviors, particularly in the contexts of sex,^12^ and body mass index (BMI),^13^ which have provided the most contradictory results in the context of whether females or males are characterized by higher ON thoughts and behaviors,^3,14,15^ or intensity of ON thoughts and behaviors increases or decreases with BMI.^13–16^

Additionally, limited studies suggest a direct influence of mindfulness on problematic eating behaviors, including ON thoughts and behaviors.^17,18^ The practice of mindfulness techniques related to eating behaviors is called ME.^19^ ME is described as sustained attention to the sensory elements of the eating experience (e.g., taste) and nonjudgmental or nonevaluative awareness of thoughts and feelings that are incongruent with the sensory elements of the current eating experience.^20^ ME helps to gradually shift external motivations for eating to internal motivations, such as hunger, thereby promoting healthier eating behaviors.^19^ So far, over a dozen studies have investigated the associations between ON thoughts and behaviors and ME.^13,17–25^ However, five of these studies used only the DOS questionnaire,^13,18–20,22^ while the other six studies used only ORTO-R,^23,24^ ORTO-15,^21^ ORTO-11,^25^ ONI,^20^ or TOS.^17^ Therefore, it would be valuable to compare the results obtained from all ORTO questionnaires with a generally accepted scale, such as PL-DOS. These considerations provide a basis for further research in this area, with a more focused sample of mindfulness specific to eating, particularly in the context of diet quality, which holds considerable relevance to ON thoughts and behaviors.

Individuals who exhibit ON thoughts and behaviors follow a rigorous diet and avoid foods containing preservatives, dyes, flavors, and products high in fat, salt, sugar, pesticides, or genetically modified foods.^26^ The list of acceptable foods may vary from person to person, but the progressive nature of the imposed eating restrictions is typical.^27^ Meal preparation and timing are also strictly controlled.^28^ Any violation of self-imposed eating rules can result in a desire to discipline oneself by increasing restrictions or fasting.^27^ Although the eating restrictions associated with a fixation on healthy eating usually lead to an improved diet, they can intensify over time, resulting in the exclusion of entire food groups.^29^ To date, one cross-sectional,^27^ and one prepost repeated cross-sectional^26^study have evaluated the diet quality of individuals exhibiting ON thoughts and behaviors. Both confirmed positive associations between ON thoughts and behaviors and the intensity of beneficial dietary characteristics for health (“Pro-Healthy Diet Index”—pHDI) and negative associations with the intensity of harmful dietary characteristics for health (“Non-Healthy Diet Index”—nHDI).^26,27^ However, in the prepost repeated cross-sectional study, these associations were observed only at the beginning of the study and not at its conclusion.^26^ Moreover, both studies used the ORTO-6 questionnaire to evaluate ON thoughts and behaviors.^26,27^

Therefore, the authors’ study aimed to (a) assess the intensity of ON thoughts and behaviors by biological sex and BMI groups; (b) evaluate the associations between ON thoughts and behaviors, ME, and diet quality; and (c) compare ORTO-R with ORTO-15 and its shortened versions (ORTO-12/−11/−9/−7) and PL-DOS to determine which ORTO questionnaire best describes ON thoughts and behaviors in the context of ME and diet quality. To the best of the authors’ knowledge, no study has simultaneously evaluated these associations using the most commonly used questionnaire for ON thoughts and behaviors (ORTO-15), its shorter versions (ORTO-12, ORTO-11, ORTO-9, ORTO-7), its new and modified version (ORTO-R), and a completely different, adapted, and validated questionnaire (PL-DOS). The authors also propose the following hypotheses:

- **H1**. Females, compared to males, exhibit a higher intensity of ON thoughts and behaviors, a higher level of ME, and a higher diet quality.
- **H2**. BMI determines the level of ME and diet quality, but does not determine ON thoughts and behaviors.
- **H3**. People with a higher intensity of ON thoughts and behaviors are characterized by a lower level of ME.
- **H4**. The diet of people with a higher intensity of ON thoughts and behaviors is characterized by higher quality.
- **H5**. ORTO-R is most closely related to PL-DOS and is the most reliable tool among all ORTO questionnaires.

## Materials and Methods

### Study Design and Participant Recruitment

**Fig. 1:**
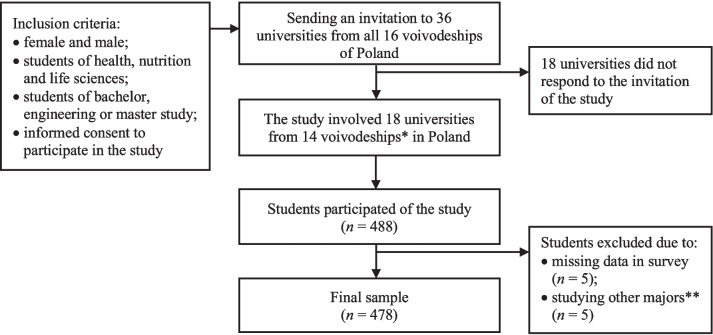
Study design and participant recruitment. * Pomerania, West Pomerania, Kuyavia-Pomerania, Podlaskie, Masovian, Lubusz, Lodzkie, Lublin, Swietokrzyskie, Subcarpathia, Lower Silesia, Opole, Silesia, Lesser Poland; ** Energetics, Navigation and ship armament, Finance and accounting, and Polish philology

A cross-sectional quantitative study was conducted between March and November 2021 among college students. Although this type of exploratory study does not establish causality, it is an appropriate first step in identifying some associations. The study protocol was approved by the Ethics Committee of the Faculty of Human Nutrition and Consumer Science, Warsaw University of Life Sciences (Resolution number 45/2017). Thirty-six universities from all sixteen voivodeships of Poland were invited to participate in the study. The selection of universities was made non-randomly. The selected universities offered majors in their academic programs related to health, nutrition, or life sciences. This selection was made due to the fact that college students of health, nutrition, and life sciences have a similar program in the early stages of their education, and all acquire knowledge in biochemistry, analytical and organic chemistry, instrumental analysis techniques, and key biological phenomena and processes occurring in the human body. Eighteen universities from fourteen voivodeships of Poland responded to the invitation and participated in the study. The computer-assisted web interview (CAWI) technique was used to conduct the study. The online survey was created using the Google Forms application. Data were collected during lectures and workshops by academic teachers from participating universities. Additionally, the invitation to the study was published on the universities’ websites, allowing students visiting these sites to also participate. Respondents were informed about the purpose, procedure, and duration of the study, as well as their right to withdraw at each stage without any consequences. Participation in the study was voluntary and anonymous. Students completed the survey in approximately 20 min. The final sample consisted of 478 students. In the preliminary analysis, ten students were excluded from further analysis due to missing data in questionnaires (*n* = 5) or not studying majors in health, nutrition, or life sciences (*n* = 5). Figure 1 presents details of the sample collection.

### Orthorexic Thoughts and Behaviors

Orthorexic thoughts and behaviors were evaluated using the validated Polish versions of the ORTO and PL-DOS questionnaires (Table 1). Firstly, the ORTO-15 questionnaire,^30^ consisting of 15 items scored on a four-point Likert scale (always, often, sometimes, never), was utilized. The coding scheme was inverted from the original ORTO-15 questionnaire by the standard procedures to facilitate easier comparison with other tools measuring ON thoughts and behaviors, for example, item two was scored as follows: 1—always, 2—often, 3—sometimes, 4—never (Appendix 1 Table 7). Many researchers have also employed shortened versions of the ORTO-15 questionnaire, such as ORTO-12,^31^ ORTO-11,^32^ ORTO-9,^33^ and ORTO-7.^34^ These versions were included in the current study, with the inverted coding scheme applied consistently. Higher scores across these questionnaires indicate a higher intensity of ON thoughts and behaviors.

**Table 1:** Internal consistency and modifications of questionnaires for assessing orthorexic thoughts and behaviors

Questionnaires of orthorexic thoughts and behaviors*	Items removed from the original ORTO-15**	Total score ranges	Averaged score ranges	Cronbach’s alpha in the total sample	Cronbach’s alpha for females	Cronbach’s alpha for males
ORTO-15	–	15–60	1–4	0.23	0.24	0.18
ORTO-12	5, 6, 8	12–48	1–4	0.18	0.22	0.13
ORTO-11	5, 8, 14, 15	11–44	1–4	0.31	0.30	0.37
ORTO-9	1, 2, 8, 9, 13, 15	9–36	1–4	0.56	0.53	0.68
ORTO-7	2, 5, 6, 8, 10, 12, 14, 15	7–28	1–4	0.03	0.04	0.02
ORTO-R	1, 2, 5, 6, 8, 9, 13, 14, 15	6–30	1–5	0.71	0.70	0.77
PL-DOS	–	10–40	1–4	0.80	0.80	0.80

^*^ ORTO-12/−11/−9/and −7 are shortened versions of the original ORTO-15 questionnaire, based on item removal; ** The item number refers to the specific item from the original ORTO-15 questionnaire; Higher Cronbach’s alpha coefficient indicates better internal consistency

Secondly, the ORTO-R questionnaire, composed of six items scored on a five-point Likert scale (never, rarely, sometimes, very often, always), was used.^10,11^ Higher scores reflect more ON thoughts and behaviors. The ORTO-R was specifically developed to address the limitations of the original ORTO-15 questionnaire and its shortened versions.^35^

Thirdly, the PL-DOS questionnaire consists of 10 items with response options as follows: 1—this does not apply to me, 2—this does rather not apply to me, 3—this does somewhat apply to me, and 4—this applies to me. A higher score indicates stronger ON thoughts and behaviors.^36^ Scores for each questionnaire were calculated by summing the scores for individual items and dividing them by the number of items. In the case of ORTO-15 and its shorter versions, the averaging of scores concerned the reversed results, while for ORTO-R and PL-DOS, it concerned the raw scores.

### Mindful Eating

The mindful eating was assessed using the Mindful Eating Scale (MES). The original version of the MES, developed by Hulbert-Williams et al.,^37^ contained 28 items, while the Polish version used in this study includes 17 items.^38^ The Polish MES consists of three subscales: act with awareness (9 items; e.g., “I eat automatically without being aware of what I am eating”), awareness (4 items; e.g., “I notice how my food looks”), and acceptance (4 items; e.g., “I tell myself I should not be hungry”). ON thoughts and behaviors, although driven by a desire for healthy eating, may paradoxically be associated with rigid eating rules and poor psychological flexibility around food; therefore, assessing their relationship with mindful eating, which emphasizes awareness and flexibility in eating behaviors, seems important from the point of view of possible countermeasures ON thought and behaviors.^13,17,22^ The original coding and scoring procedure was followed in this study.^38^ Participants rated each statement on a scale from 1 (rarely/never) to 4 (usually/always), and item scores were averaged for the raw scores (range: 1–4), with higher scores indicating a higher level of ME. Cronbach’s alpha coefficients for the MES in this study were 0.74 for act with awareness, 0.59 for awareness, and 0.80 for acceptance in the total sample; 0.74, 0.58, and 0.81 for females; and 0.72, 0.69, and 0.64 for males, respectively.

### Diet Quality

The food frequency consumption data were assessed using the Dietary Habits and Nutrition Beliefs Questionnaire (KomPAN) for individuals aged 15–65 years, developed by the Committee of Human Nutrition, Polish Academy of Sciences.^39^ The KomPAN includes key food groups in the Polish diet, namely grain products (4 items); fruit, vegetables, legumes, and potatoes (4 items); dairy products (4 items); meat, fish, and eggs (5 items); fats (2 items); beverages (3 items); sweets; and other products (2 items). These 24 food items are divided into two categories: (1) food groups with a potentially beneficial influence on health and (2) food groups with a potentially negative influence on health. Therefore, the assessment of diet quality measured by KomPAN may provide insight into whether ON thoughts and behaviors are associated with truly healthy eating behaviors or primarily reflect obsessive eating behaviors without corresponding healthy benefits.^2^. Each respondent reported their habitual consumption of food products over the past 12 months on a six-point scale ranging from 1 (never) to 6 (a few times a day). These categories were converted into daily frequencies expressed as times per day to obtain semiquantitative data: 1—0.00, 2—0.06, 3—0.14, 4—0.50, 5—1.00, and 6—2.00.^40^

Three diet indexes were subsequently created: (1) the Pro-Healthy Diet Index (pHDI), which covers 10 food groups potentially beneficial to health; (2) the Non-Healthy Diet Index (nHDI), which includes 14 food groups potentially harmful to health; and (3) the Diet Quality Index (DQI), based on the consumption of all 24 food groups included in the pHDI and nHDI. Each index was expressed as times per day and standardized to a score range of 0–100 points for pHDI and nHDI, and – 100–100 points for DQI using the following formulas:^40^

- pHDI (in points) = (100/20) × sum of the frequencies of consumption of 10 food groups (times/day),
- nHDI (in points) = (100/28) × sum of the frequencies of consumption of 14 food groups (times/day),
- DQI (in points) = (100/20) × sum of frequencies of 10 food groups (times/day) + (− 100/28) × sum of frequencies of 14 food groups (times/day).

Based on these scores, participants were categorized into three groups: 0–33 points (pHDI—low intensity of beneficial dietary characteristics for health; nHDI—low intensity of harmful dietary characteristics for health), 34–66 points (medium intensity), and 67–100 points (high intensity). For the DQI, scores ranging from − 100 to − 26 indicated a high intensity of nonhealthy dietary characteristics, − 25 to 25 indicated a low intensity of both nonhealthy and prohealthy dietary characteristics, and 26 to 100 indicated a high intensity of prohealthy dietary characteristics. In this study, Cronbach’s alpha coefficients for the KomPAN were 0.77 for the total sample, 0.79 for females, and 0.76 for males.

### Sociodemographic Characteristics

The sociodemographic characteristics of participants were assessed using questions regarding biological sex (female or male), age (in years), level of study (bachelor’s and engineering or master’s degree), year of study, major of study, university name, place of residence (village, town with 100,000 citizens or fewer, or city with more than 100,000 citizens), and voivodeship. To simplify data analysis, voivodeships were grouped into macroregions, which were divided into seven units based on the classification by Statistics Poland^41^: Northern (Pomerania, Kuyavia-Pomerania), Northwestern (West Pomerania, Lubusz), Masovian, Central (Lodzkie, Swietokrzystkie), Southwestern (Lower Silesia, Opole), Southern (Silesia, Lesser Poland), and Eastern (Podlaskie, Lublin, Subcarpathia).

### Anthropometric Data

Body mass index (BMI) was calculated based on college students’ self-reported body weight and height and categorized according to the World Health Organization guidelines^42^: underweight (BMI < 18.5 kg/m^2^), normal weight (BMI 18.5–24.9 kg/m^2^), and overweight and obesity (BMI ≥ 25.0 kg/m^2^).

### Statistical Analysis

Categorical variables were presented as percentages of the sample (%), while continuous variables were expressed as averages, and standard deviations. The normality of the distribution of continuous variables was assessed using the normal probability plot, and homogeneity of variance was tested using the *F* test and Brown–Forsythe test. Differences in average values between two independent samples with a normal distribution were analyzed using the Student’s *t*-test (for example, the differences in ORTO-15/−12/−11/−7/PL-DOS scores between females and males), whereas the *U*-Mann–Whitney test was applied for samples with an abnormal distribution (ORTO-9/-R scores between females and males). For multiple independent samples, one-way analysis of variance (ANOVA) and the Scheffe post hoc test were used for normal distributions (ORTO and PL-DOS scores between BMI groups both in females and males), while the Kruskal–Wallis Rank ANOVA and multiple comparisons of mean ranks were applied for abnormal distributions (pHDI, nHDI, and DQI scores between BMI groups in males). Associations between continuous variables were analyzed using Pearson’s correlation coefficient for normal distributions (correlation between ORTO-15 and other ORTO and PL-DOS in females) and Kendall’s tau correlation coefficient for abnormal distributions (correlation between ORTO-15 and other ORTO and PL-DOS in males). The Pearson chi-squared test was employed to determine associations between independent categorical variables, such as biological sex, BMI groups, levels of pHDI, nHDI, and DQI. Statistical analyses were performed using Statistica software version 13.3 PL (StatSoft Inc., Tulsa, OK, USA; StatSoft, Krakow, Poland).

Effect sizes and post hoc power analyses were calculated using G*Power software version 3.1.9.7 (Heinrich-Heine-Universität Düsseldorf, Düsseldorf, Germany). Different effect sizes were applied based on the statistical tests used: Cohen’s d coefficient (*d*) for the Student’s *t*-test, Glass’s rank bivariate correlation coefficient (rg) for the *U*-Mann–Whitney test, Cramer’s V coefficient (V) for the Pearson chi-squared test, eta squared (η^2^) for the one-way analysis of variance (ANOVA), and epsilon squared (ε^2^) for Kruskal–Wallis Rank ANOVA. Cohen’s d coefficient equals 0.20, indicating a small effect; equals 0.50, indicating a medium effect; and equals 0.80, indicating a large effect. Glass’s rank bivariate correlation coefficient and Cramer’s V coefficient equal 0.10, indicating a small effect; 0.30—a medium effect; and 0.50—a large effect. Eta squared and epsilon squared equal 0.01, indicating a small effect; 0.06—a medium effect; and 0.14—a large effect.^43^ Further, the value of β is the probability of making an error of the II type, meaning not rejecting the null hypothesis, which is false. The power of a test can be defined as the complement of the probability of making a type II error (β), i.e., 1-β. It was assumed that the power of the test should be at least 0.80 to ensure the detection of differences and avoid errors of the II type.^44^

The two analyses did not achieve a power of 0.80, such as the differences between the average pHDI score and both sexes (1-β err prob = 0.62), and the average pHDI score and BMI groups in females (0.66), which means that for these analyses, the risk of type II error is higher, limiting their interpretation.

## Results

### Sample Characteristics

The final sample consisted of 478 college students, including 420 (87.9%) females and 58 (12.1%) males. The average age of the students was 22.3 ± 3.1 years. Health and nutrition majors were represented by 58.6% of the participants, including fields such as food technology, human nutrition, dietetics, public health, technology and organization of gastronomy, chemical and food analytics, and quality management and food analysis. The remaining 41.4% were students of life science majors, such as biology, microbiology, chemistry, and biotechnology. Most students (75.2%) were enrolled in bachelor’s and engineering degree programs. The majority resided in the central macroregion of Poland (32.6%). The average Body Mass Index (BMI) was 22.3 ± 4.1 kg/m^2^, with males having significantly higher BMI compared to females (*p* < 0.0001; rg = 0.72) (Table 2).

**Table 2:** Sociodemographic characteristics of the study sample across biological sex groups

Variables	Total sample	Female	Male	U/Chi^2^	Df	V/rg	*p*-value
	*N* = 478	*N* = 420	*N* = 58				
Age in years (M ± SD)	22.3 ± 3.1	22.3 ± 2.9	22.5 ± 4.1	− 0.01^●^	476	0.06^▲^	0.997
Place of residence *N* (%)							
Village	154 (32.2)	140 (33.3)	14 (24.1)	2.37^●●^	2	0.07^▲▲^	0.305
Town ≤ 100.000 citizens	105 (22.0)	89 (21.2)	16 (27.6)				
City > 100.000 citizens	219 (45.8)	191 (45.5)	28 (48.3)				
Macroregion *N* (%)							
Northern	42 (8.8)	34 (8.1)	8 (13.8)	15.48^●●^	6	0.18^▲▲^	**0.017**
North-western	64 (13.4)	50 (11.9)	14 (24.1)				
Masovian	32 (6.7)	31 (7.4)	1 (1.7)				
Central	156 (32.6)	134 (31.9)	22 (37.9)				
South-western	73 (15.3)	68 (16.2)	5 (8.6)				
Southern	40 (8.4)	36 (8.6)	4 (6.9)				
Eastern	71 (14.8)	67 (15.9)	4 (6.9)				
Level of study *N* (%)							
Bachelor and engineering studies	364 (75.2)	319 (75.9)	45 (77.6)	0.07^●●^	1	0.01^▲▲^	0.784
Master studies	114 (24.8)	101 (24.1)	13 (22.4)				
College major *N* (%)							
Health and nutrition	280 (58.6)	248 (59.1)	32 (55.2)	0.23^●●^	1	− 0.03^▲▲^	0.574
Life science	198 (41.4)	172 (40.9)	26 (44.8)				
BMI in kg/m^2^ (M ± SD)	22.3 ± 4.1	21.9 ± 3.8	25.1 ± 5.0	5.25^●^	476	0.72^▲^	** < 0.0001**
BMI categories *N* (%)							
Underweight	61 (12.8)	55 (13.1)	6 (10.3)	18.78^●●^	2	0.19^▲▲^	** < 0.0001**
Normal weight	327 (68.4)	298 (70.9)	29 (50.0)				
Overweight	90 (18.8)	67 (15.9)	23 (39.7)				

N—number of participants; %—sample percentage; M—average; SD—standard deviation; Df—degrees of freedom; BMI—body mass index; Significance set at *p* < 0.05; ^●^U-Mann Whitney test (U); ^●●^Pearson chi-squared test (Chi^2^); ^▲^Glass’s rank bivariate correlation coefficient (rg); ^▲▲^Cramer’s V coefficient (V)

### Orthorexic Thoughts and Behaviors, Mindful Eating, and Diet Quality Across Biological Sex and BMI

The average scores for all ORTO questionnaires were similar, ranging from 2.3 ± 0.4 to 2.7 ± 0.7, while the lowest score was observed for the PL-DOS questionnaire (2.0 ± 0.5). Females exhibited a higher intensity of orthorexic thoughts and behaviors compared to males, but this difference was significant only for PL-DOS (*p* < 0.001; *d* = 0.60). The average ME scores for the three subscales were as follows: 3.1 ± 0.5 for act with awareness, 3.2 ± 0.5 for awareness, and 3.0 ± 0.8 for acceptance. A majority of individuals (80.6%) exhibited low intensity of both nonhealthy and prohealthy dietary characteristics, as indicated by the DQI. Compared to males, females demonstrated higher pHDI (*p* = 0.015; rg = 0.28) and DQI scores (*p* < 0.001; rg = 0.50), whereas males exhibited higher nHDI scores than females (*p* = 0.008; rg = 0.43). The effect sizes of these results ranged from moderate to large. No significant differences in ME scores for any of the three subscales were observed across biological sex groups. None of the evaluated students achieved high nHDI scores; therefore, this information was omitted from the tables below (Table 3).

**Table 3:** Orthorexic thoughts and behaviors, mindful eating, and diet quality in the total sample and biological sex groups

Variables	Total sample	Female	Male	U/t/Chi^2^	Df	V/d/rg	*p*-value
	*N* = 478	*N* = 420	*N* = 58				
Orthorexic thoughts and behaviors (M ± SD)							
ORTO-15 score	2.6 ± 0.3	2.6 ± 0.3	2.6 ± 0.3	− 0.03^●●●^	476	0.00^▲▲▲^	0.973
ORTO-12 score	2.6 ± 0.3	2.6 ± 0.3	2.6 ± 0.3	− 0.11^●●●^	476	0.00^▲▲▲^	0.913
ORTO-11 score	2.6 ± 0.3	2.6 ± 0.3	2.5 ± 0.4	− 0.36^●●●^	476	0.28^▲▲▲^	0.716
ORTO-9 score	2.6 ± 0.4	2.6 ± 0.4	2.5 ± 0.5	− 1.64^●^	93	0.22^▲^	0.099
ORTO-7 score	2.3 ± 0.4	2.3 ± 0.4	2.3 ± 0.4	0.79^●●●^	476	0.00^▲▲▲^	0.429
ORTO-R score	2.7 ± 0.7	2.8 ± 0.7	2.5 ± 0.9	− 1.57^●^	93	0.37^▲^	0.115
PL-DOS score	2.0 ± 0.5	2.1 ± 0.5	1.8 ± 0.5	− 3.58^●●●^	476	0.60^▲▲▲^	** < 0.001**
Mindful eating (M ± SD)							
Act with awareness	3.1 ± 0.5	3.2 ± 0.5	3.1 ± 0.5	− 0.83^●^	93	0.20^▲^	0.407
Awareness	3.2 ± 0.5	3.2 ± 0.5	3.3 ± 0.5	1.64^●●●^	476	0.20^▲▲▲^	0.101
Acceptance	3.0 ± 0.8	3.0 ± 0.8	3.2 ± 0.7	1.35^●●●^	476	0.27^▲▲▲^	0.179
pHDI score (M ± SD)	27.9 ± 13.6	28.4 ± 13.6	24.7 ± 13.1	− 2.44^●^	93	0.28^▲^	**0.015**
Level pHDI *N* (%)							
Low	334 (69.9)	287 (68.3)	47 (81.0)	10.83^●●^	2	0.15^▲▲^	**0.004**
Medium	140 (29.3)	131 (31.2)	9 (15.5)				
High	4 (0.8)	2 (0.5)	2 (3.5)				
nHDI score (M ± SD)	15.4 ± 9.0	14.9 ± 8.6	19.1 ± 11.0	2.65^●^	93	0.43^▲^	**0.008**
Level of nHDI *N* (%)							
Low	462 (96.7)	410 (97.6)	52 (89.7)	9.99^●●^	1	− 0.14^▲▲^	**0.002**
Medium	16 (3.3)	10 (2.4)	6 (10.3)				
DQI score (M ± SD)	12.5 ± 15.4	13.5 ± 14.9	5.6 ± 16.5	− 3.48^●^	93	0.50^▲^	** < 0.001**
Level of DQI *N* (%)							
High intensity of nHDI	2 (0.4)	0 (0.0)	2 (3.5)	17.37^●●^	2	0.19^▲▲^	** < 0.001**
Low intensity of nHDI and pHDI	385 (80.6)	335 (79.8)	50 (86.2)				
High intensity of pHDI	91 (19.0)	85 (20.2)	6 (10.3)				

N—number of participants; %—sample percentage; M–average; SD—standard deviation; pHDI—Pro-Healthy Diet Index; nHDI—Non-Healthy Diet Index; DQI—Diet-Quality Index; Significance set at *p* < 0.05; ^●^U-Mann Whitney test (U); ^●●^Pearson chi-squared test (Chi^2^); ^●●●^t-Student’s test for independent variables (t); ^▲^Glass’s rank bivariate correlation coefficient (rg); ^▲▲^ Cramer's V coefficient (V); ^▲▲▲^Cohen’s d coefficient (d); Higher scores of all ORTO and PL-DOS reflect higher intensity of ON thoughts and behaviors

Females with underweight exhibited a lower intensity of ON thoughts and behaviors compared to those with normal weight and overweight, as measured by ORTO-R (*p* = 0.008; η^2^ = 0.16). Acceptance, as an element of ME, was lower among females with overweight compared to those with underweight and normal weight (*p* < 0.0001; ε^2^ = 0.24). Females with normal weight demonstrated higher pHDI (*p* = 0.029; η^2^ = 0.13) and DQI scores (*p* = 0.006; η^2^ = 0.17) compared to those with underweight. The effect sizes of these results are moderate or large. No significant differences were observed in pHDI, nHDI, and DQI levels or nHDI scores across BMI groups (Table 4).

**Table 4:** Orthorexic thoughts and behaviors, mindful eating, and diet quality in females across the BMI groups

Variables	Total sample	Underweight	Normal weight	Overweight	H/F/Chi^2^	V/η^2^/ε^2^	Df	*p*-value
	*N* = 420	*N* = 55	*N* = 298	*N* = 67				
Orthorexic thoughts and behaviors (M ± SD)								
ORTO-15 score	2.6 ± 0.3	2.5 ± 0.3	2.6 ± 0.3	2.5 ± 0.2	2.54^●^	0.15^▲^	2	0.080
ORTO-12 score	2.6 ± 0.3	2.6 ± 0.3	2.6 ± 0.3	2.5 ± 0.3	1.91^●^	0.12^▲^	2	0.149
ORTO-11 score	2.6 ± 0.3	2.5 ± 0.3	2.6 ± 0.3	2.5 ± 0.3	3.08^●^	0.15^▲^	2	0.050
ORTO-9 score	2.6 ± 0.4	2.5 ± 0.4	2.7 ± 0.4	2.6 ± 0.4	1.99^●^	0.18^▲^	2	0.138
ORTO-7 score	2.3 ± 0.4	2.3 ± 0.4	2.3 ± 0.4	2.2 ± 0.4	1.20^●^	0.09^▲^	2	0.303
ORTO-R score	2.8 ± 0.7	2.5 ± 0.8^a^	2.8 ± 0.7^b^	2.9 ± 0.7^b^	4.92^●^	0.16^▲^	2	**0.008**
PL-DOS score	2.1 ± 0.5	1.9 ± 0.5	2.1 ± 0.5	2.1 ± 0.5	2.99^●^	0.13^▲^	2	0.051
Mindful eating (M ± SD)								
Act with awareness	3.2 ± 0.5	3.2 ± 0.5	3.2 ± 0.5	3.1 ± 0.5	1.08^●^	0.07^*▲*^	2	0.341
Awareness	3.2 ± 0.5	3.3 ± 0.5	3.2 ± 0.5	3.2 ± 0.4	0.37^●^	0.07^*▲*^	2	0.689
Acceptance	3.0 ± 0.8	3.3 ± 0.7^a^	3.0 ± 0.8^a^	2.6 ± 0.8^b^	24.54^●●^	0.24^*▲▲*^	2	** < 0.0001**
pHDI score (M ± SD)	28.4 ± 13.6	24.1 ± 12.8^a^	29.3 ± 13.7^b^	27.6 ± 13.4^a,b^	3.58^●^	0.13^*▲*^	2	**0.029**
Level pHDI *N* (%)								
Low	287 (68.3)	45 (81.8)	194 (65.1)	48 (71.6)	6.88^●●●^	0.09^*▲▲▲*^	4	0.141
Medium	131 (31.2)	10 (18.2)	102 (34.2)	19 (28.4)				
High	2 (0.5)	0 (0.0)	2 (0.7)	0 (0.0)				
nHDI score (M ± SD)	14.9 ± 8.6	16.2 ± 8.9	14.6 ± 8.6	15.2 ± 8.7	0.88^●^	0.06^*▲*^	2	0.416
Level of nHDI *N* (%)								
Low	410 (97.6)	53 (96.4)	292 (97.9)	65 (97.0)	0.65^●●●^	0.04^*▲▲▲*^	2	0.722
Medium	10 (2.4)	2 (3.6)	6 (2.0)	2 (2.9)				
DQI score (M ± SD)	13.5 ± 14.9	7.9 ± 12.8^a^	14.8 ± 15.5^b^	12.3 ± 13.2^a,b^	5.19^●^	0.17^*▲*^	2	**0.006**
Level of DQI *N* (%)								
Low intensity of nHDI and pHDI	335 (79.8)	50 (90.9)	230 (77.2)	55 (82.1)	5.69^●●●^	0.12^*▲▲▲*^	2	0.058
High intensity of pHDI	85 (20.2)	5 (9.1)	68 (22.8)	12 (17.9)				

N—number of participants; %—sample percentage; M—average; SD—standard deviation; Df—degrees of freedom; pHDI—Pro-Healthy Diet Index; nHDI—Non-Healthy Diet Index; DQI—Diet-Quality Index; ^●^One-way analysis of variance (ANOVA; F), and Scheffe test; ^●●^Kruskal–Wallis Rank ANOVA test (H), and multiple comparisons of mean ranks for all samples; ^a,b^means differ statistically significantly at *p* < 0.05; ^●●●^Chi-squared test (Chi^2^); ^▲^eta squared (η^2^); ^▲▲^epsilon squared (ε^2^); ^▲▲▲^Cramer’s V coefficient (V); Higher scores of all ORTO and PL-DOS reflect higher intensity of ON thoughts and behaviors

Males, when divided into BMI groups, did not show significant differences in ON thoughts and behaviors, ME, or diet quality (Table 5).

**Table 5:** Orthorexic thoughts and behaviors, mindful eating, and diet quality in males across the BMI groups

Variables	Total sample	Underweight	Normal weight	Overweight	H/F/Chi^2^	V/η^2^/ε^2^	Df	*p*-value
	*N* = 58	*N* = 6	*N* = 29	*N* = 23				
Orthorexic thoughts and behaviors (M ± SD)								
ORTO-15 score	2.6 ± 0.3	2.5 ± 0.3	2.6 ± 0.2	2.6 ± 0.3	0.16^●^	0.10^▲^	2	0.850
ORTO-12 score	2.6 ± 0.3	2.6 ± 0.3	2.6 ± 0.2	2.6 ± 0.3	0.05^●^	0.00^▲^	2	0.950
ORTO-11 score	2.5 ± 0.4	2.5 ± 0.5	2.5 ± 0.3	2.6 ± 0.4	0.12^●^	0.12^▲^	2	0.886
ORTO-9 score	2.5 ± 0.5	2.3 ± 0.7	2.5 ± 0.4	2.6 ± 0.5	0.73^●^	0.18^▲^	2	0.487
ORTO-7 score	2.3 ± 0.4	2.3 ± 0.4	2.4 ± 0.4	2.3 ± 0.4	0.10^●^	0.13^▲^	2	0.353
ORTO-R score	2.5 ± 0.9	2.5 ± 1.4	2.5 ± 0.7	2.6 ± 0.9	0.21^●^	0.05^▲^	2	0.813
PL-DOS score	1.8 ±.05	1.5 ± 0.6	1.9 ± 0.5	1.9 ± 0.5	1.44^●^	0.24^▲^	2	0.245
Mindful eating (M ± SD)								
Act with awareness	3.1 ± 0.5	3.0 ± 0.6	3.1 ± 0.4	3.1 ± 0.6	0.18^●^	0.06^▲^	2	0.837
Awareness	3.3 ± 0.5	3.6 ± 0.4	3.2 ± 0.5	3.4 ± 0.4	2.18^●^	0.33^▲^	2	0.122
Acceptance	3.2 ± 0.7	3.3 ± 0.7	3.2 ± 0.6	3.0 ± 0.8	0.59^●^	0.16^▲^	2	0.558
pHDI score (M ± SD)	24.7 ± 13.1	24.2 ± 23.2	25.8 ± 12.9	23.5 ± 10.0	1.80^●●^	0.07^▲▲^	2	0.406
Level pHDI *N* (%)								
Low	47 (81.0)	5 (83.3)	23 (79.3)	19 (82.6)	4.89^●●●^	0.21^▲▲▲^	4	0.298
Medium	9 (15.5)	0 (0.0)	5 (17.2)	4 (17.4)				
High	2 (3.5)	1 (16.7)	1 (3.5)	0 (0.0)				
nHDI score (M ± SD)	19.1 ± 11.0	21.9 ± 12.0	17.9 ± 12.6	19.9 ± 8.7	3.32^●●^	0.12^▲▲^	2	0.190
Level of nHDI *N* (%)								
Low	52 (89.7)	5 (83.3)	25 (86.2)	22 (95.7)	1.52^●●●^	0.16^▲▲▲^	2	0.467
Medium	6 (10.3)	1 (16.7)	4 (13.8)	1 (4.4)				
DQI score (M ± SD)	5.6 ± 16.5	2.2 ± 28.2	7.9 ± 14.9	3.6 ± 15.0	1.90^●●^	0.12^▲▲^	2	0.386
Level of DQI *N* (%)								
High intensity of nHDI	2 (3.5)	0 (0.0)	1 (3.5)	1 (4.4)	1.74^●●●^	0.12^▲▲▲^	4	0.783
Low intensity of nHDI and pHDI	50 (86.2)	5 (83.3)	24 (82.8)	21 (91.3)				
High intensity of pHDI	6 (10.3)	1 (16.7)	4 (13.8)	1 (4.4)				

N—number of participants; %—sample percentage; M–average; SD—standard deviation; Df—degrees of freedom; pHDI—Pro-Healthy Diet Index; nHDI—Non-Healthy Diet Index; DQI—Diet-Quality Index; ^●^One-way analysis of variance (ANOVA; F), and Scheffe test; ^●●^Kruskal–Wallis Rank ANOVA test (H), and multiple comparisons of mean ranks for all samples; ^a,b^means differ statistically significantly at *p* < 0.05; ^●●●^Chi-squared test (Chi^2^); ^▲^eta squared (η^2^); ^▲▲^epsilon squared (ε^2^); ^▲▲▲^Cramer’s V coefficient (V); Higher scores of all ORTO and PL-DOS reflect higher intensity of ON thoughts and behaviors

### Association Between Orthorexic Thoughts and Behaviors, Mindful Eating, and Diet Quality

ORTO-15 and its shortened versions correlated at least highly positively with each other (*p* < 0.001; r/τ ≥ 0.567 for all), and similar correlations were found between ORTO-R and PL-DOS for both sexes (*p* < 0.001; r ≥ 0.653 for all). Whereby, the ORTO-R and PL-DOS exhibited at most moderate positive correlations with ORTO-15, −12, −11, and −7 for females and/or males (*p* < 0.05; r/τ ≥ 0.275 for all). ORTO-R and PL-DOS showed the most consistent correlations with mindful eating aspects, in contrast to the ORTO-15 questionnaire and its shortened versions. A negligible negative correlation was observed between ORTO-R and act with awareness (*p* < 0.05; τ = − 0.091), while PL-DOS showed a weak negative correlation with awareness for females (*p* < 0.0001; *r* = –0.111). At least, moderate negative correlations were found between ORTO-R, PL-DOS, and acceptance for both sexes (*p* < 0.05; *r* ≥ –0.336 for all). An increase in the intensity of ON thoughts and behaviors measured by almost all questionnaires was associated with an increase in the intensity of beneficial dietary characteristics for health (pHDI and DQI scores) and a decrease in the intensity of harmful dietary characteristics for health (nHDI scores) (Table 6).

**Table 6:** Correlations between orthorexic thoughts and behaviors, mindful eating, and diet quality among females and males

Variables	1	2	3	4	5	6	7	8	9	10	11	12	13
ORTO-15 (1)		**0.938 **^**r**^ ***	**0.922 **^**r**^ ***	**0.829 **^**r**^ ***	**0.789 **^**r**^ ***	**0.438 **^**r**^ ***	**0.412 **^**r**^ ***	**0.159 **^**τ**^ ***	**0.160 **^**r**^ **	− 0.021 ^r^	**0.111 **^**τ**^ **	**–0.114 **^**τ**^ **	**0.156 **^**τ**^ ***
ORTO-12 (2)	**0.805 **^**τ**^ ***		**0.908 **^**r**^ ***	**0.771 **^**r**^ ***	**0.827 **^**r**^ ***	**0.432 **^**r**^ ***	**0.332 **^**r**^ ***	**0.085 **^**τ**^ *	**0.166 **^**r**^ *	− 0.079 ^r^	0.061 ^τ^	–0.053 ^τ^	**0.077 **^**τ**^ *
ORTO-11 (3)	**0.827 **^**τ**^ ***	**0.914 **^**r**^ ***		**0.795 **^**r**^ ***	**0.844 **^**r**^ ***	**0.435 **^**r**^ ***	**0.354 **^**r**^ ***	**0.148 **^**τ**^ ***	**0.179 **^**r**^ **	0.002^r^	**0.139 **^**τ**^ **	–0.052 ^τ^	**0.152 **^**τ**^ ***
ORTO-9 (4)	**0.691 **^**τ**^ ***	**0.779 **^**r**^ ***	**0.833 **^**r**^ ***		**0.579 **^**r**^ ***	**0.596 **^**r**^ ***	**0.511 **^**r**^ ***	**0.075 **^**τ**^ *	**0.146 **^**r**^ **	** − 0.203 **^**r**^ ***	**0.150 **^**τ**^ ***	**–0.096 **^**τ**^ *	**0.184 **^**τ**^ ***
ORTO-7 (5)	**0.567 **^**τ**^ ***	**0.798 **^**r**^ ***	**0.826 **^**r**^ ***	**0.504 **^**r**^ ***		**0.295 **^**r**^ ***	**0.239 **^**r**^ ***	**0.083 **^**τ**^ *	**0.116 **^**r**^ *	− 0.024 ^r^	**0.069 **^**τ**^ *	–0.047 ^τ^	**0.076 **^**τ**^ *
ORTO-R (6)	**0.275 **^**τ**^ *	**0.327 **^**r**^ *	**0.398 **^**r**^ *	**0.667 **^**r**^ ***	0.130 ^r^		**0.653 **^**r**^ ***	**–0.091 **^**τ**^ *	− 0.038 ^r^	** − 0.557 **^**r**^ ***	**0.113 **^**τ**^ **	**–0.069 **^**τ**^ *	**0.138 **^**τ**^ ***
PL-DOS (7)	**0.377 **^**τ**^ ***	**0.349 **^**r**^ *	**0.389 **^**r**^ *	**0.616 **^**r**^ ***	0.217 ^r^	**0.689 **^**r**^ ***		0.005 ^τ^	** − 0.111 **^**r**^ ***	** − 0.385 **^**r**^ ***	**0.112 **^**τ**^ **	**–0.149 **^**τ**^ ***	**0.172 **^**τ**^ ***
Act with awareness (8)	**0.301 **^**τ**^ **	**0.217 **^**τ**^ *	**0.296 **^**τ**^ *	0.145 ^τ^	**0.239 **^**τ**^ **	–0.051 ^τ^	0.098 ^τ^		**0.096 **^**τ**^ *	**0.326 **^**τ**^ ***	**0.073 **^**τ**^ *	**–0.189 **^**τ**^ ***	**0.178 **^**τ**^ ***
Awareness (9)	0.069 ^τ^	0.195 ^r^	0.189 ^r^	0.035 ^r^	0.233 ^r^	0.056 ^r^	–0.044 ^r^	0.149 ^τ^		**0.102 **^**τ**^ *	**0.114 **^**τ**^ ***	0.001 ^τ^	**0.108 **^**τ**^ **
Acceptance (10)	0.092 ^τ^	0.153 ^r^	0.204 ^r^	–0.147 ^r^	**0.296 **^**r**^ *	**–0.518 **^**r**^ ***	**–0.336 **^**r**^ *	**0.349 **^**τ**^ **	0.118 ^r^		0.019 ^τ^	–0.043 ^τ^	0.046 ^τ^
pHDI score (11)	**0.268 **^**τ**^ *	**0.206 **^**τ**^ *	**0.295 **^**τ**^ *	**0.231 **^**τ**^ *	**0.201 **^**τ**^ *	**0.121 **^**τ**^	**0.262 **^**τ**^ *	**0.304 **^**τ**^ **	0.063 ^τ^	0.165 ^τ^		0.057 ^τ^	**0.635 **^**τ**^ ***
nHDI score (12)	–0.119 ^τ^	–0.001 ^τ^	–0.069 ^τ^	–0.106 ^τ^	–0.026 ^τ^	–0.083 ^τ^	–**0.247 **^**τ**^ *	–0.175 ^τ^	0.147 ^τ^	0.133 ^τ^	0.015 ^τ^		**–0.309 **^**τ**^ ***
DQI score (13)	**0.308 **^**τ**^ **	**0.193 **^**τ**^ *	**0.312 **^**τ**^ **	**0.278 **^**τ**^ *	**0.186 **^**τ**^ *	0.148 ^τ^	**0.379 **^**τ**^ ***	**0.398 **^**τ**^ ***	− 0.050 ^τ^	0.046 ^τ^	**0.514 **^**τ**^ ***	**–0.472 **^**τ**^ ***	

Correlations for females are presented above the main diagonal, while correlations for males are presented below the main diagonal; *p < 0.05; **p < 0.001; ***p < 0.0001; ^r^ Pearson’s correlation coefficient; ^τ^ tau Kendall’s correlation coefficient; pHDI—Pro-Healthy Diet Index; nHDI—Non-Healthy Diet Index; DQI—Diet-Quality Index; Significance set at *p* < 0.05; Higher scores of all ORTO and PL-DOS reflect higher intensity of ON thoughts and behaviors

## Discussion

### Comparing Questionnaires

This study evaluated the associations between orthorexic thoughts and behaviors, ME, and diet quality by biological sex and BMI groups. It also compared ORTO-R with ORTO-15 and its shortened versions (ORTO-12/−11/−9/−7) and PL-DOS to determine which ORTO questionnaire best describes ON thoughts and behaviors in the context of ME and diet quality. In this study, ON thoughts and behaviors were analyzed on a continuous scale for all questionnaires. This approach avoided the methodological flaws of the ORTO-15, such as applying “unsafe” thresholds to diagnose ON,^10^ and enabled a direct comparison between questionnaires. Two distinct groups of questionnaires emerged from the analysis. The first group included ORTO-15 and its shortened versions, including ORTO-12, −11, −9, and −7. The second group included ORTO-R and PL-DOS. It is worth noting that of all ORTO questionnaires, only ORTO-9 correlated highly positively with ORTO-R and PL-DOS, which seems surprising given its high positive correlations with ORTO-15 and the rest of its shortened versions. Nevertheless, this result may be due to the fact that the same items were removed in ORTO-R and partially in ORTO-9. A recent meta-analysis was conducted to obtain a more accurate overall reliability coefficient estimate and investigate the reliability coefficient among the various adaptations of the ORTO questionnaires in all populations and language versions.^45^ The study showed that ORTO-15 and its shortened versions (ORTO-12, −11, −9, and −7) achieved a minimum reliability coefficient of 0.23 and a maximum reliability coefficient of 0.83, which suggests a low-reliability coefficient, showing that the reliability and dependability of the ORTO questionnaires are low and statistical errors are highly possible.^45^ In addition, ORTO-R has achieved a reliability coefficient in the range of 0.70–0.79.^45^ Similarly, in this study, the internal consistency of the questionnaires for assessing ON thoughts and behaviors was found to be acceptable only for the ORTO-R and PL-DOS (Cronbach’s alpha coefficients more than 0.70), whereby for the ORTO-15 and its shortened versions these coefficients ranged from 0.03 to 0.56 for the total sample. Therefore, it may be beneficial to use multiple questionnaires to assess ON thoughts and behaviors. Applying a combination of tools provides a more comprehensive and reliable understanding of ON, allowing for a broader assessment of its various aspects and reducing biases or limitations associated with relying on a single questionnaire.

### Orthorexic Thoughts and Behaviors, and Mindful Eating

Previous studies on ON thoughts and behaviors and mindfulness have generally reported negative correlations between the two constructs.^17,20–23^ However, this study found positive correlations between ON thoughts and behaviors and ME for almost all ORTO questionnaires. Analysis of the MES questionnaire subscales revealed that ORTO-15, −12, −11, −9, and −7 positively correlated with act with awareness and awareness, with a slight exception for ORTO-9, which negatively correlated with acceptance, but only for females. Similarly, in males, positive correlations were observed between ORTO-15, −12, −11, −7, and act with awareness, as well as between ORTO-7 and acceptance. Although one previous study also found a positive correlation between ON thoughts and behaviors and ME, it used ORTO-11.^25^ The explanation for this can be found in the fact that some components of mindful eating, such as increased nutritional awareness, analyzing the body’s reactions to food, or choosing foods with the intention of maintaining health, may resemble behaviors characteristic of orthorexia, although without their obsessive and rigid nature.^21 ^For this reason, the positive correlation between mindful eating and ON thoughts and behaviors as measured by ORTO-15 or its shortened versions may result not so much from the actual co-occurrence of these phenomena as from methodological limitations of the measurement questionnaire and common behavioral features of both constructs.^21^ Therefore, it is necessary to interpret the results with caution and to use additional tools that allow for a distinction between adaptive and pathological concern for healthy eating. In contrast, the findings for ORTO-R and PL-DOS were consistent with prior studies. ORTO-R negatively correlated with act with awareness, while PL-DOS negatively correlated with awareness for females. Additionally, both ORTO-R and PL-DOS negatively correlated with acceptance for both sexes. These findings align with previous studies in the context of awareness^13,19,22^ and acceptance,^17^ both of which are essential components of ME.^46^ The negative correlations between ON thoughts and behaviors and ME observed with ORTO-R and PL-DOS suggest that individuals practicing ME are less likely to exhibit high-intensity ON thoughts and behaviors.^23^ This aligns with the conceptualization of ME as promoting an unrestricted and compassionate approach to eating, potentially alleviating the strict eating restrictions associated with ON thoughts and behaviors.^23^ Moreover, ME may not only moderate ON thoughts and behaviors but also provide protective effects against the pathology of eating disorders.^17^

### Orthorexic Thoughts and Behaviors, Biological Sex, and BMI

In terms of ME, ON thoughts and behaviors, BMI, and sex, several notable findings emerged. In this study, females with underweight and normal weight exhibited higher acceptance compared to those with overweight. Previous studies have confirmed associations between ME and BMI,^47,48^ showing that ME decreases as BMI increases.^47^ These findings suggest that interventions focused on ME may help prevent not only eating disorders but also obesity. In the current study, females with underweight exhibited a lower intensity of ON thoughts and behaviors compared to those with normal weight and overweight. Additionally, females exhibited a higher intensity of ON thoughts and behaviors than males. Studies exploring associations between ON thoughts and behaviors, BMI, and sex have yielded mixed results.^1,12,13^ Consistent with the findings of Oberle et al.^16^, higher BMI was associated with greater intensity of ON thoughts and behaviors. Conversely, other studies have reported reversed associations^13^or no associations between BMI and ON thoughts and behaviors.^22,49^ Regarding sex, some researchers observed a higher intensity of ON thoughts and behaviors in females than males, which was confirmed in the current study.^15,49^ Although certain studies have reported a higher intensity of ON thoughts and behaviors in males than females,^3^ as well as the lack of any associations between sex and ON thoughts and behaviors.^14^ These findings highlight the complex nature of ON thoughts and behaviors and their associations with BMI and sex, emphasizing the need for further studies to understand cultural and psychological differences.

### Orthorexic Thoughts and Behaviors, and Diet Quality

Moreover, females in this study exhibited higher diet quality than males. Generally, studies indicate that females demonstrate better diet quality,^50,51^ potentially because they often bear most of the cooking responsibilities at home,^52^ prioritize healthy diets and experience greater social pressure associated with dietary choices.^53^ In this context, it is also necessary to mention ON thoughts and behaviors, the characteristic of which is fixation on the diet quality. In this study, ON thoughts and behaviors were associated with better diet quality, including increased intensity of beneficial/healthy dietary characteristics for health (higher pHDI/DQI scores) and reduced intensity of harmful dietary characteristics for health (lower nHDI scores). Previous studies confirm these findings, associating ON thoughts and behaviors with better diet quality.^26,27^ It was observed that ON thoughts and behaviors are associated with higher pHDI^26,27^and DQI,^26^ while lower nHDI.^26,27^ This indicates that individuals with a higher intensity of ON thoughts and behaviors tend to reduce their intake of unhealthy foods. However, it is crucial to note that ON thoughts and behaviors are associated with a compulsive fixation on healthy eating.^1^ While an initially healthy diet may have benefits, it can devolve into a deficient diet with significant negative medical consequences.^27^ It is worth adding that almost all questionnaires assessing ON thoughts and behaviors in this study correlated with indexes of diet quality with similar direction and strength, making it difficult to compare these questionnaires with each other. Perhaps the use of a different tool for assessing diet quality would have allowed for a greater variety of results, potentially highlighting distinctions between the questionnaires.

### Strengths and Limitations

The strength of the authors’ study lies in the relatively diverse sample of Polish students from eighteen universities located across all seven macroregions of Poland. In addition, the students attended varied courses of study within health and nutrition and life sciences. The use of the CAWI technique ensured respondent anonymity, facilitating candid responses to sensitive questions.^54^ Furthermore, to the best of the authors’ knowledge, this is the first study to evaluate the associations between orthorexic thoughts and behaviors, ME, and diet quality using different versions of the ORTO questionnaires alongside the PL-DOS questionnaire.

Nevertheless, the authors’ study has several limitations. The data were gathered exclusively from students in food, nutrition, and life sciences, which limits the generalizability of the findings to the broader student population. Furthermore, only 12.1% of the sample were males, indicating that male representation was insufficient, which severely limits the conclusions that can be drawn regarding biological sex. Moreover, only six males were underweight, which probably determined the lack of differences between BMI groups and the assessed characteristics. Self-reported weight and height were used to calculate BMI, which may introduce inaccuracies, as the validity of self-reported data can be influenced by sociodemographic factors such as biological sex.^55^ Moreover, two analyses did not achieve adequate power, i.e., for differences between the average pHDI score and both biological sexes and between the average pHDI score and BMI groups in females. As a result, effect sizes for these analyses were moderate. It is worth adding that analyses comparing levels of pHDI, nHDI, and DQI and biological sex were small, which could undermine confidence in these findings. Furthermore, the non-random selection of universities was based on their accessibility, which could have caused potential selection bias. Finally, the cross-sectional design of the study, with data collected at a single point in time, precluded the ability to assess causality in the observed associations between variables.

## Implications for Behavioral Health

These findings extend the current literature by enhancing the understanding of orthorexic thoughts and behaviors as measured by various questionnaires and by describing the nature and direction of their associations with ME and diet quality. A comparison of six ORTO questionnaires (ORTO-15, −12, −11, −9, −7, -R) with the PL-DOS questionnaire revealed that ORTO-R, alongside PL-DOS, best captured ON thoughts and behaviors, especially in the context of ME. The negative correlations between each element of ME (i.e., acting with awareness, awareness, acceptance) and ORTO-R, as well as PL-DOS, suggest that ME practices may be associated with reduced ON thoughts and behaviors and warrant further investigation as a therapeutic approach (e.g., mindfulness-based programs, psychoeducation on flexible eating, and cognitive-behavioral techniques). However, the association of ON thoughts and behaviors with a better diet quality may suggest that complexity and conceptual overlap between them. As ON thoughts and behaviors are characterized by an excessive focus on healthy eating, it is perhaps unsurprising that stronger ON thoughts and behaviors correlate with higher diet quality scores-especially when diet quality is operationalized based on conventional dietary guidelines. Future research on diet quality among individuals with ON thoughts and behaviors should not only employ varied measures to assess diet quality, such as diet recalls, food diaries, or objective nutritional biomarkers, but also adopt longitudinal designs. Such approaches could help capture the potential decline in diet quality resulting from increasingly restrictive eating patterns over time.

## Supplementary Information

Below is the link to the electronic supplementary material.

Supplementary file1 (DOCX 20 KB)

## Data Availability

All the data and syntaxes used for the analyses presented within the paper are stored at the open science repository, which is available at the link: https://doi.org/10.18150/YJGWHK.
