# Supplementary material for: Comparing Questionnaires for Assessing Orthorexic Thoughts and Behaviors in College Students
Source: J Behav Health Serv Res. 2025 Jul 25;53(3):404–28. doi: 10.1007/s11414-025-09957-z (PMC13315514; doi:10.1007/s11414-025-09957-z)
Supplement: Supplementary file 1 — Supplementary file1 (DOCX 20 KB) [file 11414_2025_9957_MOESM1_ESM.docx]

Table 7. Method of coding the ORTO-15 questionnaire in the study.

| Number of items | Items | Coding of the original ORTO-15 | | | | Reversed coding of the ORTO-15 | | | |
| --- | --- | --- | --- | --- | --- | --- | --- | --- | --- |
|  |  | Always | Often | Sometimes | Never | Never | Sometimes | Often | Always |
| 1 | When eating, do you pay attention to the calories of the food? | 2 | 4 | 3 | 1 | 4 | 2 | 1 | 3 |
| 2 | When you go in a food shop do you feel confused? | 4 | 3 | 2 | 1 | 4 | 3 | 2 | 1 |
| 3 | In the last 3 months, did the thought of food worry you? | 1 | 2 | 3 | 4 | 1 | 2 | 3 | 4 |
| 4 | Are your eating choices conditioned by your worry about your health status? | 1 | 2 | 3 | 4 | 1 | 2 | 3 | 4 |
| 5 | Is taste of food more important than the quality when you evaluate food? | 4 | 3 | 2 | 1 | 4 | 3 | 2 | 1 |
| 6 | Are you willing to spend more money to have healthier food? | 1 | 2 | 3 | 4 | 1 | 2 | 3 | 4 |
| 7 | Does the thought about food worry you for more than three hours a day? | 1 | 2 | 3 | 4 | 1 | 2 | 3 | 4 |
| 8 | Do you allow yourself any eating transgressions? | 4 | 3 | 2 | 1 | 1 | 2 | 3 | 4 |
| 9 | Do you think your mood affects your eating behaviour? | 4 | 3 | 2 | 1 | 4 | 3 | 2 | 1 |
| 10 | Do you think that the conviction to eat only healthy food increases self-esteem? | 1 | 2 | 3 | 4 | 1 | 2 | 3 | 4 |
| 11 | Do you think that eating healthy food changes your life-style (frequency of eating out, friends…)? | 1 | 2 | 3 | 4 | 1 | 2 | 3 | 4 |
| 12 | Do you think that consuming healthy food may improve your appearance? | 1 | 2 | 3 | 4 | 1 | 2 | 3 | 4 |
| 13 | Do you feel guilty when transgressing? | 2 | 4 | 3 | 1 | 4 | 2 | 1 | 3 |
| 14 | Do you think that on the market there is also unhealthy food? | 1 | 2 | 3 | 4 | 1 | 2 | 3 | 4 |
| 15 | At present, are you alone when having meals? | 1 | 2 | 3 | 4 | 1 | 2 | 3 | 4 |
